# Time‐ and cost‐effective manufacturing of 3D templates of the pelvis and acetabulum in primary and revision hip arthroplasty using semiautomatic segmentation

**DOI:** 10.1002/jeo2.70547

**Published:** 2025-11-14

**Authors:** Hendrik Pott, Peter Savov, Ricarda Stauss, Julian‐Arman Beheshty, Felix Thormann, Max Ettinger, Stephan Brand

**Affiliations:** ^1^ Division of Orthopaedics at Campus Pius‐Hospital, School of Medicine and Health Sciences Carl von Ossietzky Universität Oldenburg Oldenburg Germany

**Keywords:** 3D printing, acetabular defects, efficiency, revision arthroplasty, total hip arthroplasty

## Abstract

**Purpose:**

To develop and evaluate a time‐ and cost‐efficient workflow for generating three‐dimensional (3D) printable models of the pelvis and acetabulum using semiautomated segmentation with free and open‐source software. The goal was to enable a streamlined production of individualized models suited for preoperative planning and education in primary and revision hip arthroplasty (THA).

**Methods:**

A semiautomated segmentation protocol was created using 3D Slicer and the TotalSegmentator module, followed by targeted manual refinement. This was compared to traditional manual segmentation using computed tomography (CT) data sets from patients undergoing complex primary or revision THA. The workflow was validated by two participants, who independently performed the segmentation and 3D printing process after studying the step‐by‐step guide as provided in this article for 8 native hips and 12 pathologically altered hip joints. Segmentation time, material use and print duration were recorded. A learning curve analysis was conducted via a logarithmic regression analysis.

**Results:**

The semiautomated workflow reduced segmentation time considerably compared to manual segmentation. Both investigators were able to learn and apply the workflow with increasing speed over successive trials, indicating a steep learning curve. Segmentation times amounted to a mean of less than 10 min after a learning curve of five segmentations in native hip models and eight segmentations for pathologically altered joints. The generated models were anatomically accurate and suitable for surgical planning. Cost analysis showed substantial savings compared to commercial outsourcing with mean costs of less than 5 euros for both native and pathological hip models.

**Conclusion:**

The presented workflow offers a fast, accessible, and low‐cost method to produce patient‐specific 3D models for THA planning. It can be adopted in most clinical environments using freely available software and standard 3D printers. This approach may help democratize access to 3D printing technology in orthopaedic surgery and lay the foundation for future clinical applications.

**Level of Evidence:**

Level IV.

**Abbreviations**
3D: three‐dimensional
CT: computed tomography
kWh: kilowatt‐hour
PLA: polylactic acid
THA: total hip arthroplasty

## INTRODUCTION

Three‐dimensional (3D) printing has emerged as a valuable tool in preoperative planning for total hip arthroplasty (THA), particularly in complex primary and revision cases [1, 2, 8]. Several studies have demonstrated the benefits of using 3D printed anatomical models to improve implant positioning and restore anatomical landmarks such as the hip rotation centre [5, 6, 15, 16]. These models enable detailed visualization of patient‐specific pelvic morphology, contributing to more precise surgical planning and enhanced training of surgical residents [6, 8].

Despite its clinical potential, the widespread adoption of 3D printing in orthopaedics remains limited by technical and economic barriers. Many available workflows require expensive commercial software or rely on manual segmentation, which is time‐consuming and demands significant user expertise [3, 7, 11]. A cost‐effective, time‐efficient and user‐friendly solution is needed to make 3D printing more accessible in routine clinical settings.

In this study, we present a step‐by‐step workflow for generating 3D printable templates of the pelvis and acetabulum using free and open‐source software tools, specifically 3D Slicer (https://www.slicer.org) in combination with the TotalSegmentator module for semiautomated segmentation [4, 13]. This is followed by minimal manual refinement to adjust for individual anatomical variations, such as bone loss or deformation. The workflow is designed to be reproducible, intuitive and accessible—even to users with limited prior experience in medical 3D printing.

Furthermore, we compare this semiautomated approach to traditional manual segmentation in terms of time efficiency and demonstrate that this workflow enables nonspecialist users to produce high‐quality anatomical models for surgical planning. The goal of this paper is to provide a practical and scalable solution for integrating 3D printing into clinical orthopaedic workflows without incurring substantial financial or time‐related burdens.

## MATERIALS AND METHODS

### Study population

This study included data sets of 15 patients who underwent computed tomography (CT) imaging of the pelvis in the context of either complex primary or revision THA. Imaging data were anonymized and retrospectively analysed in accordance with institutional ethical guidelines. Table 1 shows the acetabular defect types present in our patient cohort.

**Table 1:** Table showing the type of acetabular defects present in our patient cohort according to the classification by Paprosky et al. [10].

Paprosky type	Number of patients
2B	3
2C	2
3A	2
3B	5

### Study design

Two segmentation approaches were evaluated and compared:

1. Manual segmentation, performed using 3D Slicer and the ‘threshold’ and ‘grow from seeds’ features (version 5.6.2).
2. A semiautomated workflow using the TotalSegmentator module within 3D Slicer, followed by targeted manual refinement of the acetabulum to account for anatomical variations, including bone loss (version 76aaa53). TotalSegmentator is free of charge, but needs to be downloaded via the Extension Manager (View ‐>Extension Manager ‐> Search ‐> TotalSegmentator ‐> Install, Source Code: https://github.com/lassoan/SlicerTotalSegmentator).

Each method was used to generate a printable 3D model of the pelvis and acetabulum.

The first eight attempts performed by the main author (H. P.) using manual segmentation were timed. After eight manual segmentations, the semiautomated workflow was introduced. The last manual segmentation was performed again using the semiautomatic workflow. The first eight attempts for segmentations using the semiautomatic workflow were timed and compared to the manual segmentation in order to analyse the time difference between the two approaches.

After establishing the semiautomated protocol, two participants without prior experience in 3D printing were provided with a draft version of the workflow (J.‐A. B. and F. T.). First, both investigators independently performed segmentations of eight native hip joints, and then 12 pathologically altered hip joints that featured acetabular defects were analysed. The native, for example, uninjured and nonpathological, hips were identified on the contralateral side of the pathological hips within the same imaging datasets. Completed segmentations were checked for anatomical accuracy by both the main author and the respective investigator, appreciating frontal, sagittal and axial planes as well as the 3D model.

To analyse the learning curve, the time required to complete each segmentation attempt was recorded. These times were then plotted against the attempt number to visualize the learning curve. A logarithmic regression model was applied to quantify improvement over time.

Material use and printing time were recorded for each model and a cost analysis was performed considering material and power costs. Material costs were calculated by BambooLab based on the current rates for the polylactic acid (PLA) filament at the time of submission (August 2025), and an electricity rate of 0.35 euros/kWh was assumed for cost analysis.

The data was tested for normal distribution using a Shapiro–Wilk test. Paired and unpaired *t*‐tests were used to test for significance. A *p* value of <0.05 was deemed significant.

### Printing setup and parameters

All 3D models were exported as STL files and processed using Bambu Studio (BambuLab, Version 2.1.0.59). Printing was performed using the BambuLab H2D 3D printer with PLA matte filament. Key printing parameters are illustrated in Table 2.

**Table 2:** Printing parameters.

Setting	Value
Layer height/initial layer height	0.24 mm/0.2 mm
Line width	0.42 mm
Wall loops	2
Sparse infill density	15%
Sparse infill pattern	Grid

### Step‐by‐step workflow

**Figure 1:**
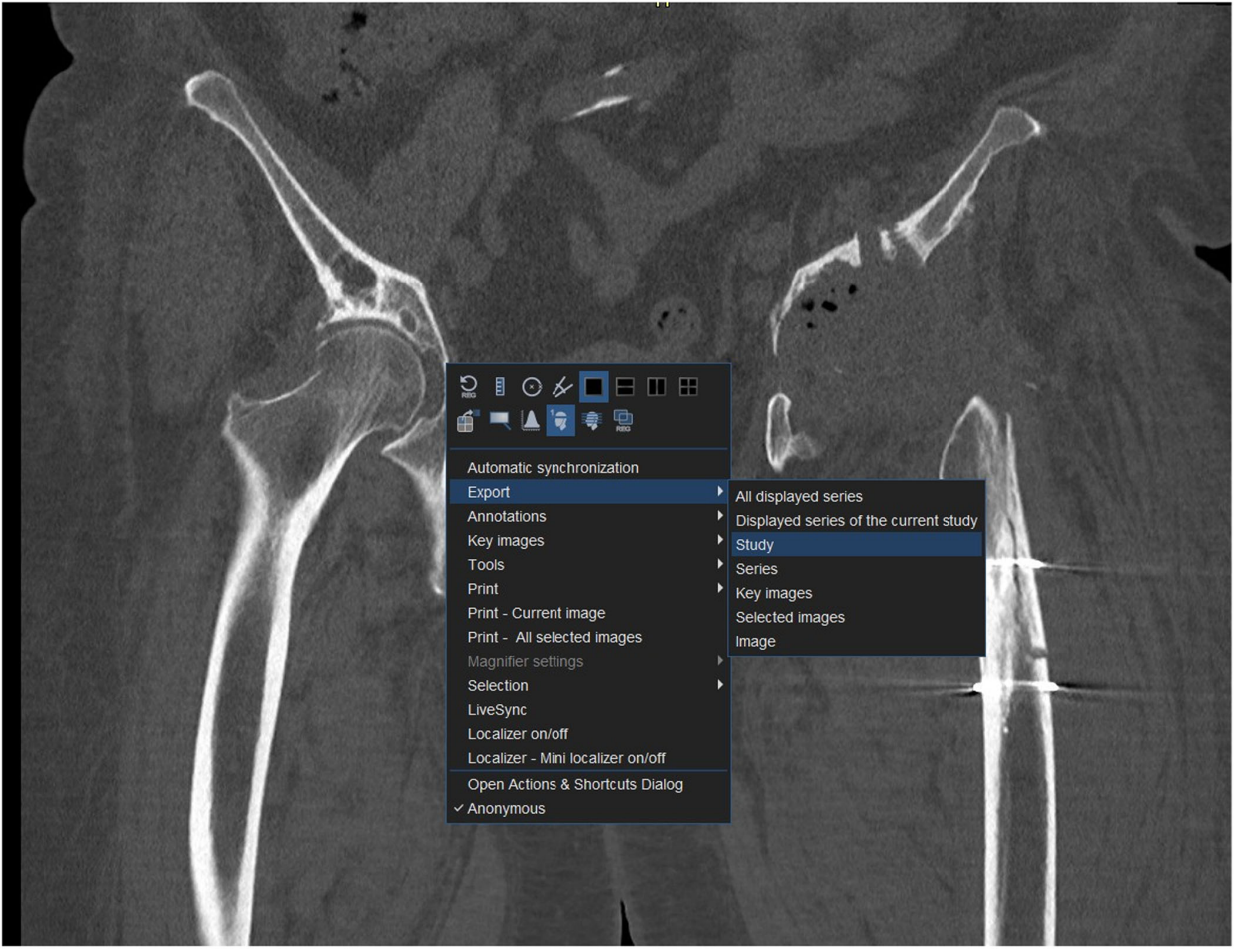
Export and save DICOM data locally using the ‘export’ command (DICOM Viewer: DeepUnity Review, Dedalus).

**Figure 2:**
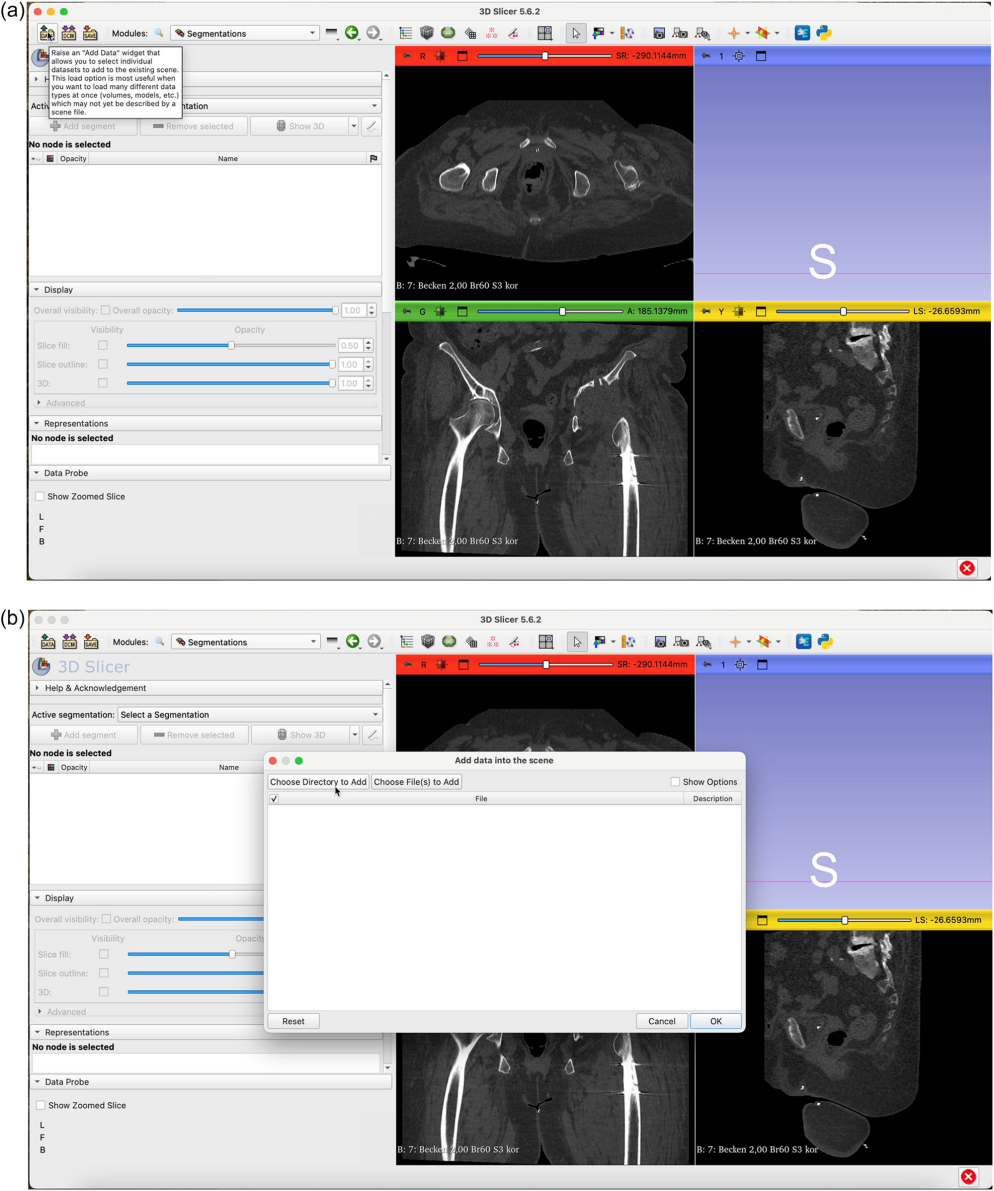
Using 3D Slicer, click the Data button (a) and select the desired directory to upload data (b).

**Figure 5:**
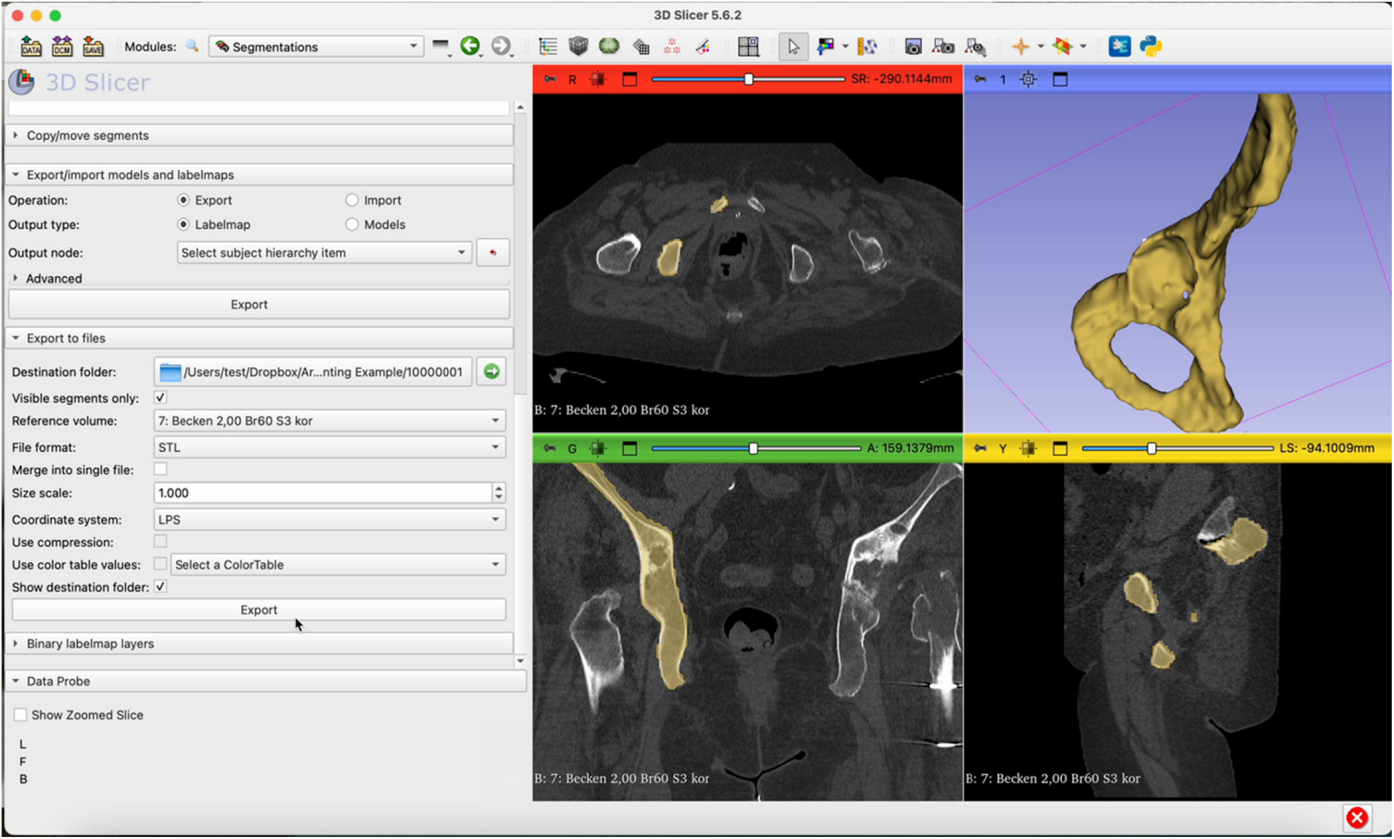
Switch to the segmentations module. Export the segmented model as an STL file to the desired folder.

The step‐by‐step workflow is explained in the following section featuring Figures 1, 2, 3, 4, 5, 6.

Figure 3Open the Total Segmentator module by clicking on the magnifying glass (a) and searching for ‘Total Segmentator’ (b). Download the plug‐in separately if necessary. Once the module is open, click Apply (c). The plug‐in will segment each organ structure individually. It is usually sufficient to use the ‘fast’ mode. This takes approximately one minute on average (d).

**Figure:**
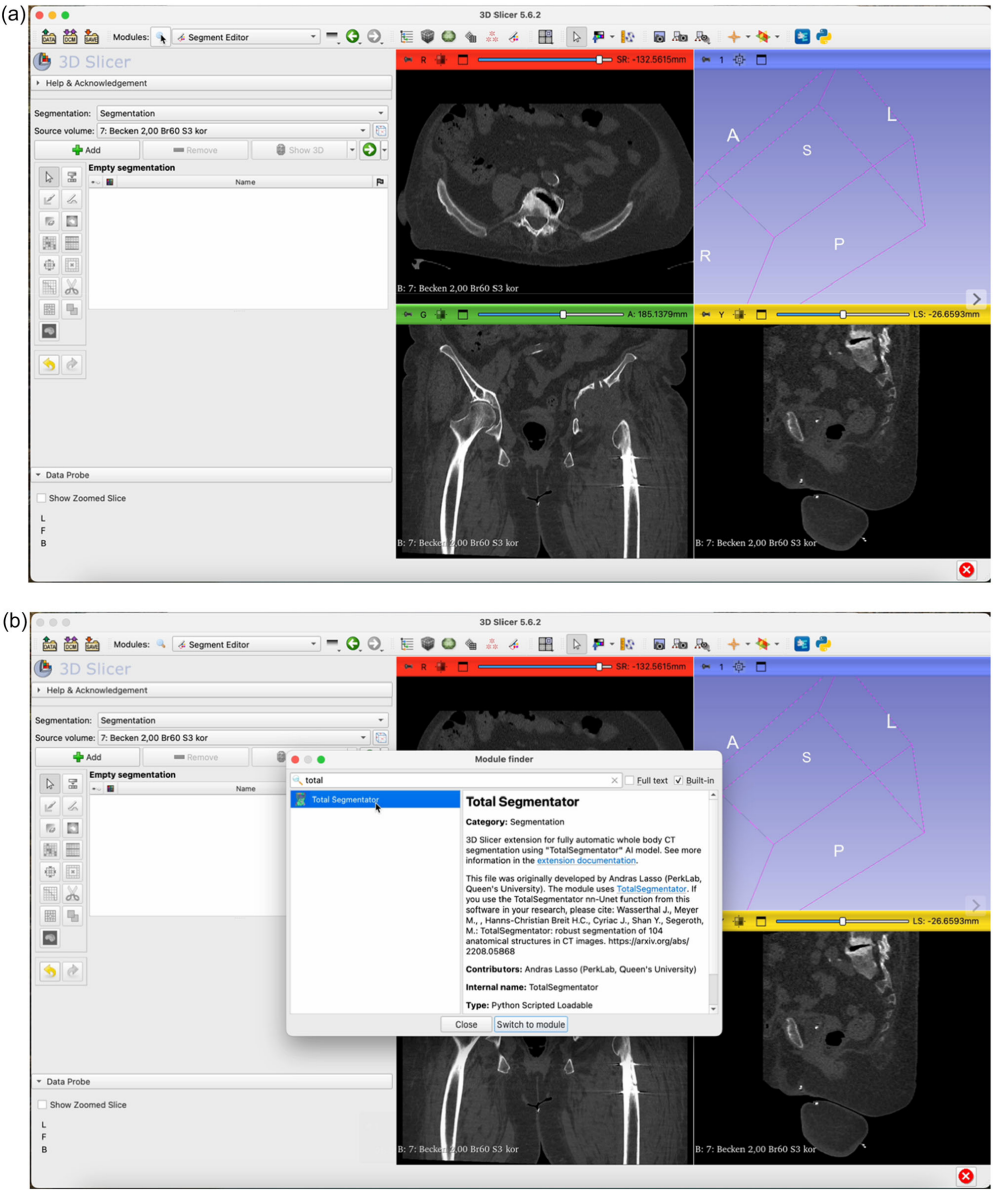

**Figure:**
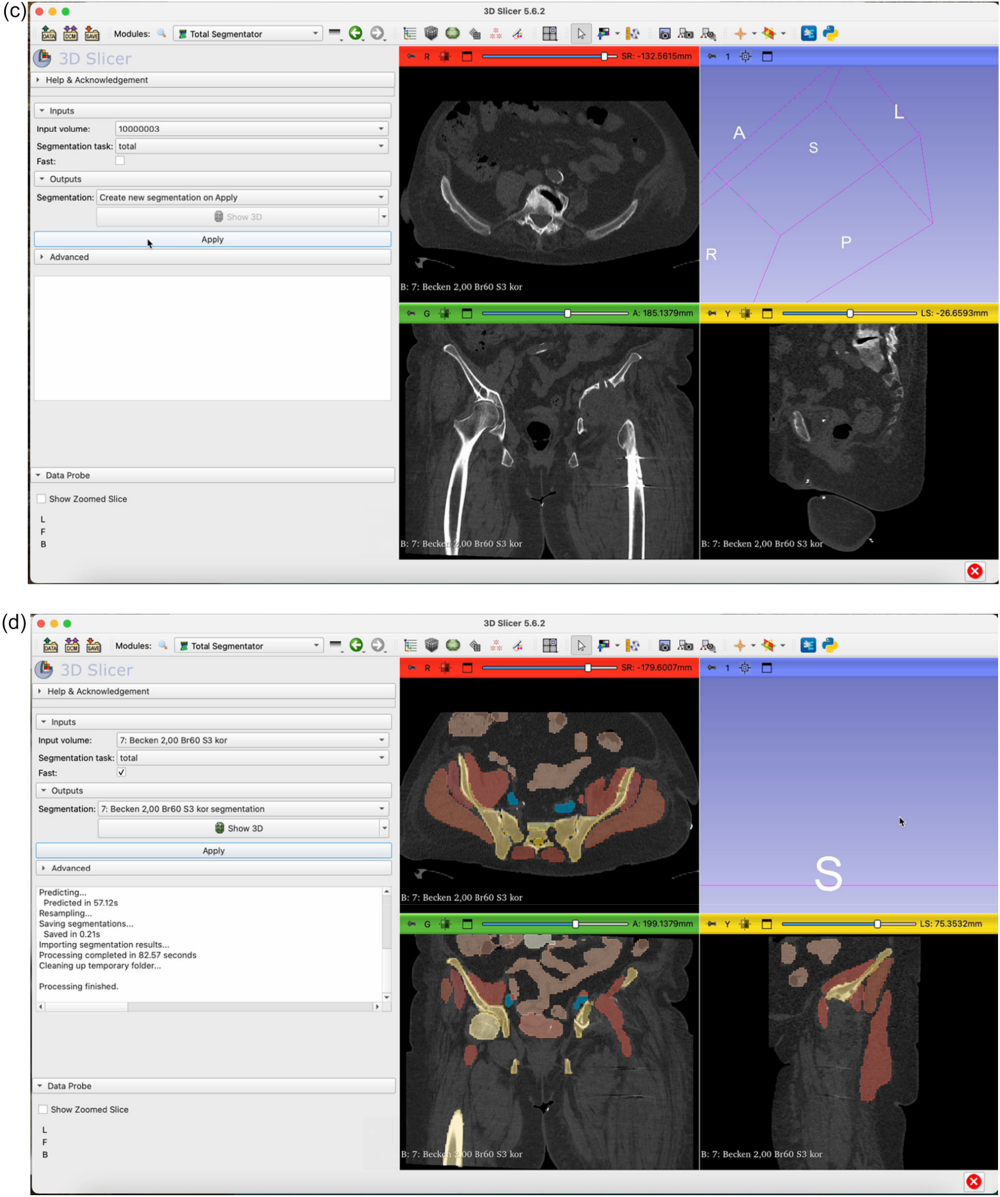

Figure 4Switch to the Segment Editor to refine the segmentation (a). Hide unneeded organ structures by clicking the eye icon next to each item (b, c, d). Use the Erase function to remove overlay from the acetabulum in all three planes of view (e–g). Use the Smoothing tool to eliminate any residual incongruencies in the acetabulum (h, i). A smoothing factor of 0.70 is often appropriate. Check all three planes as well as the three‐dimensional (3D)‐view to ensure the acetabulum is accurately represented (j).

**Figure:**
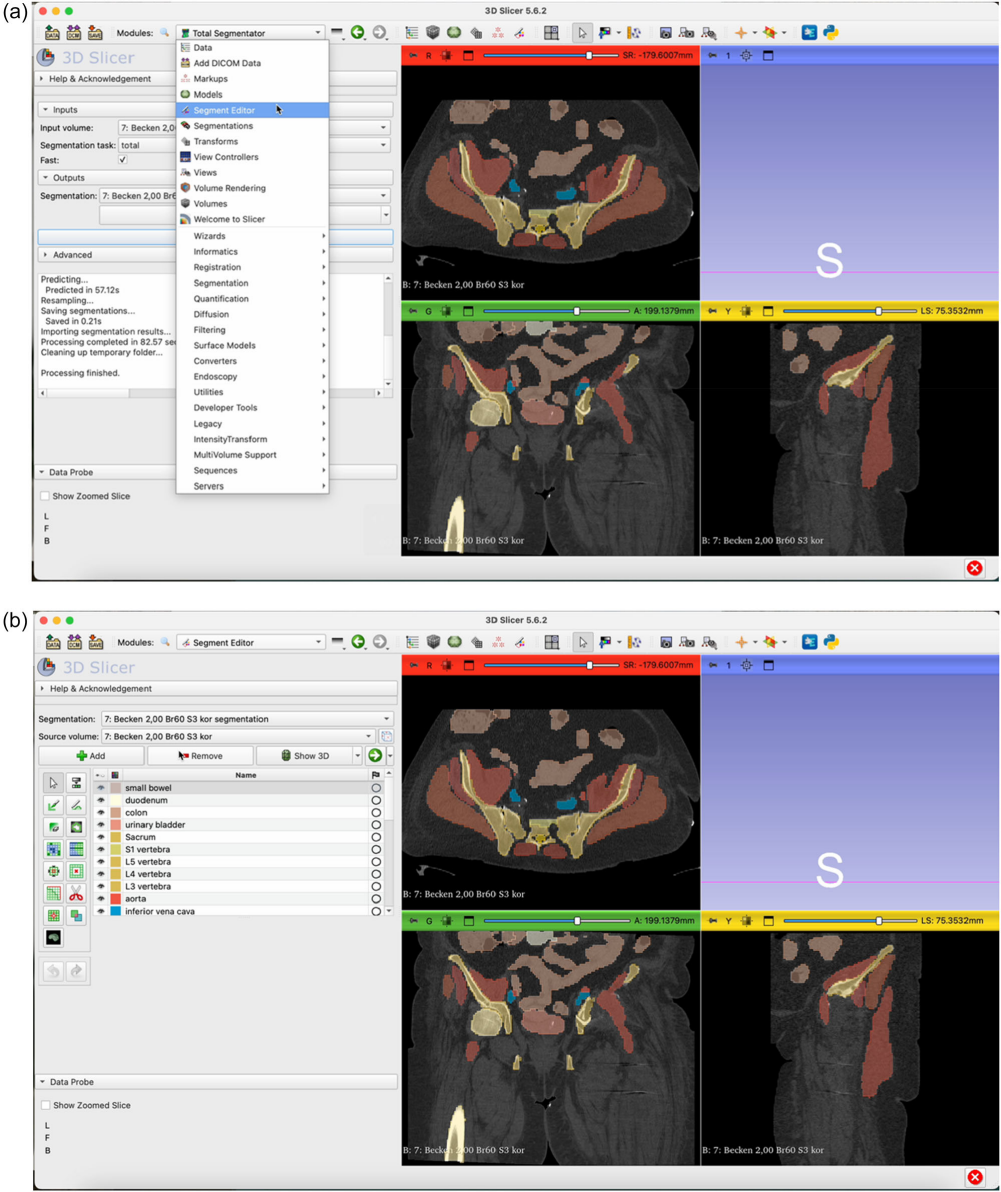

**Figure:**
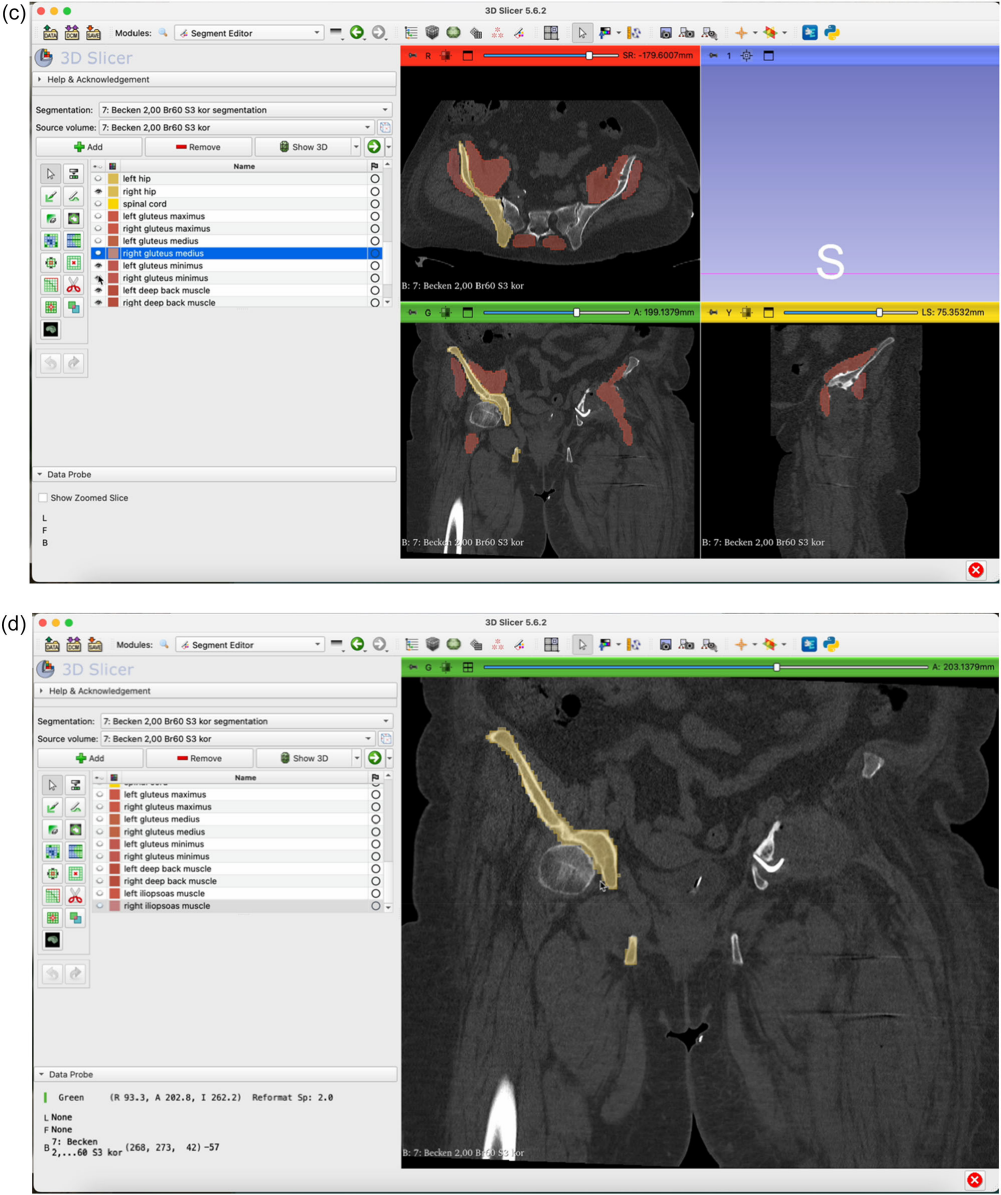

**Figure:**
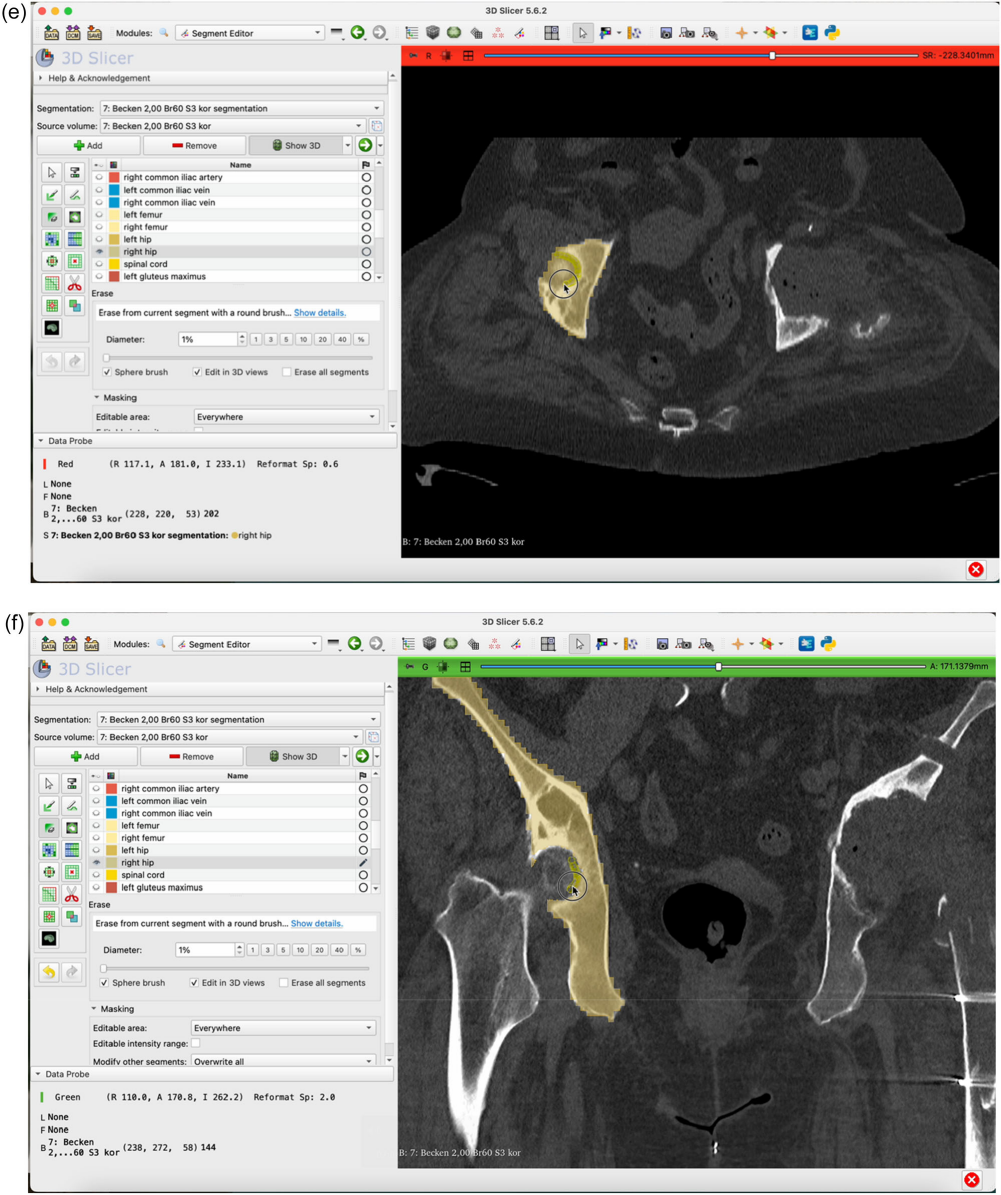

**Figure:**
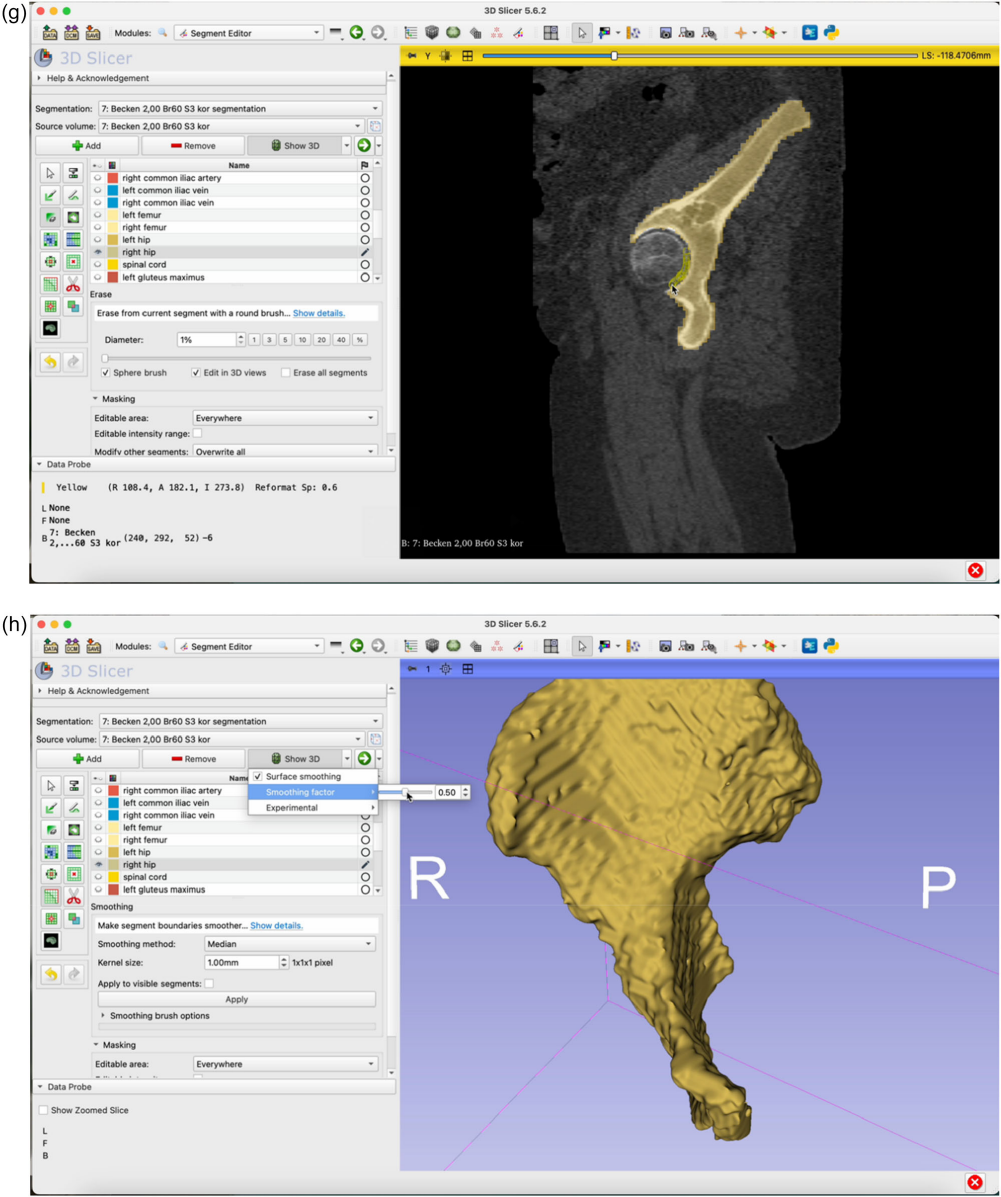

**Figure:**
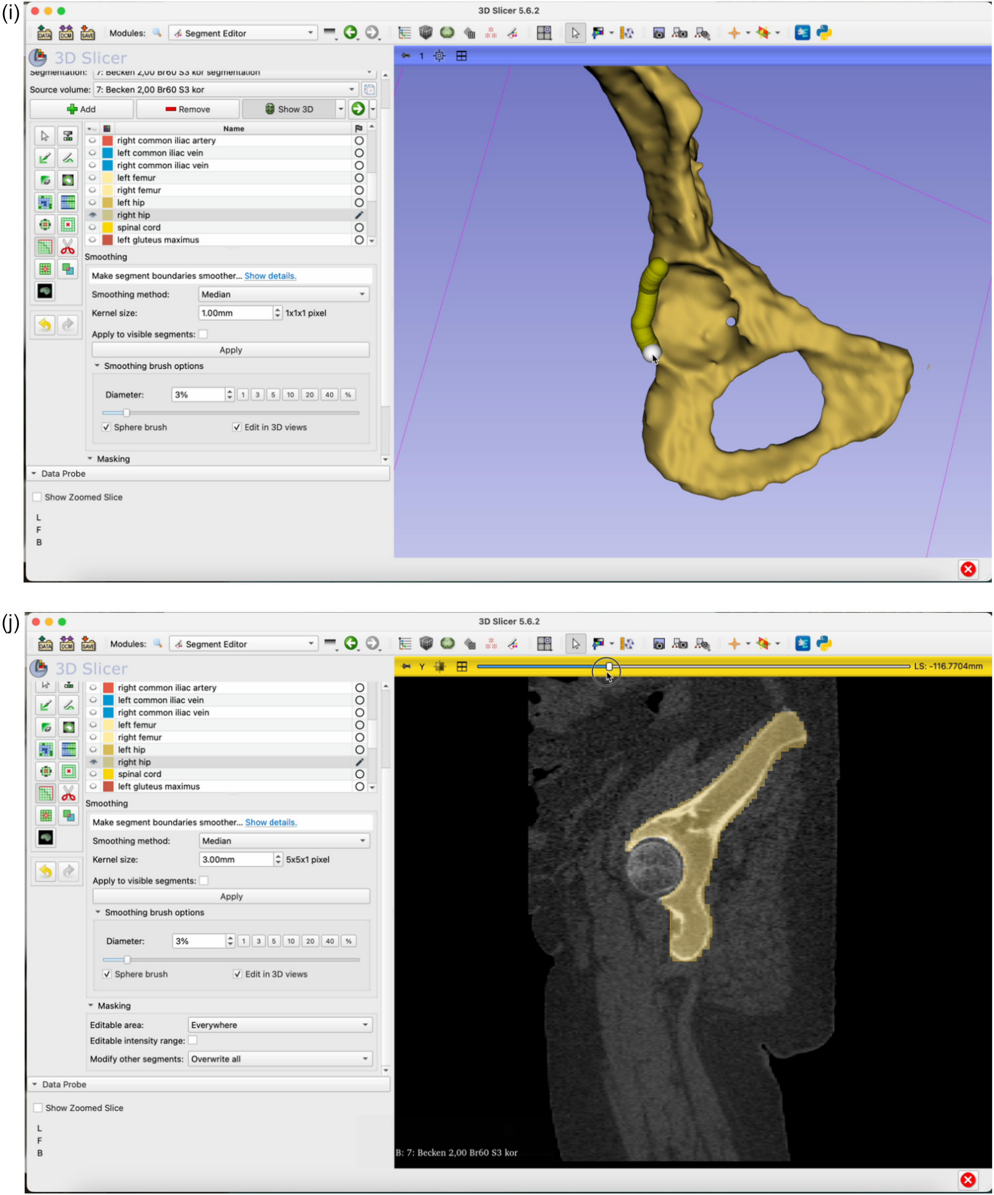

Figure 6Open the BambooLab software. Click Add Object to import the STL file into the 3D printing software (a). Click Auto‐Orient to optimize the print orientation (b). Select the Tree support type (c). Click Slice Plate and then Print Plate (d). Review the estimated filament usage, material cost and print duration provided by the software (e).

**Figure:**
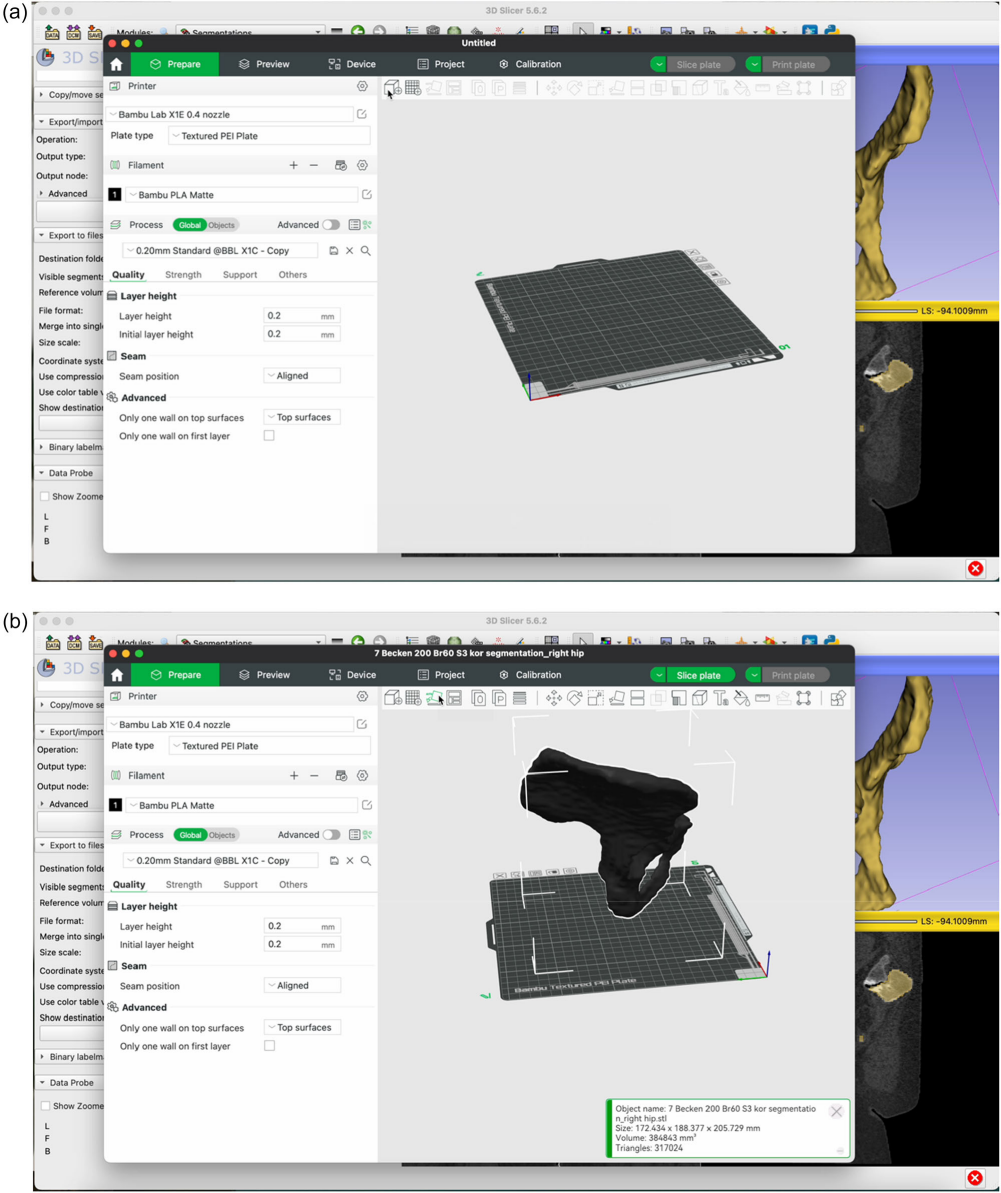

**Figure:**
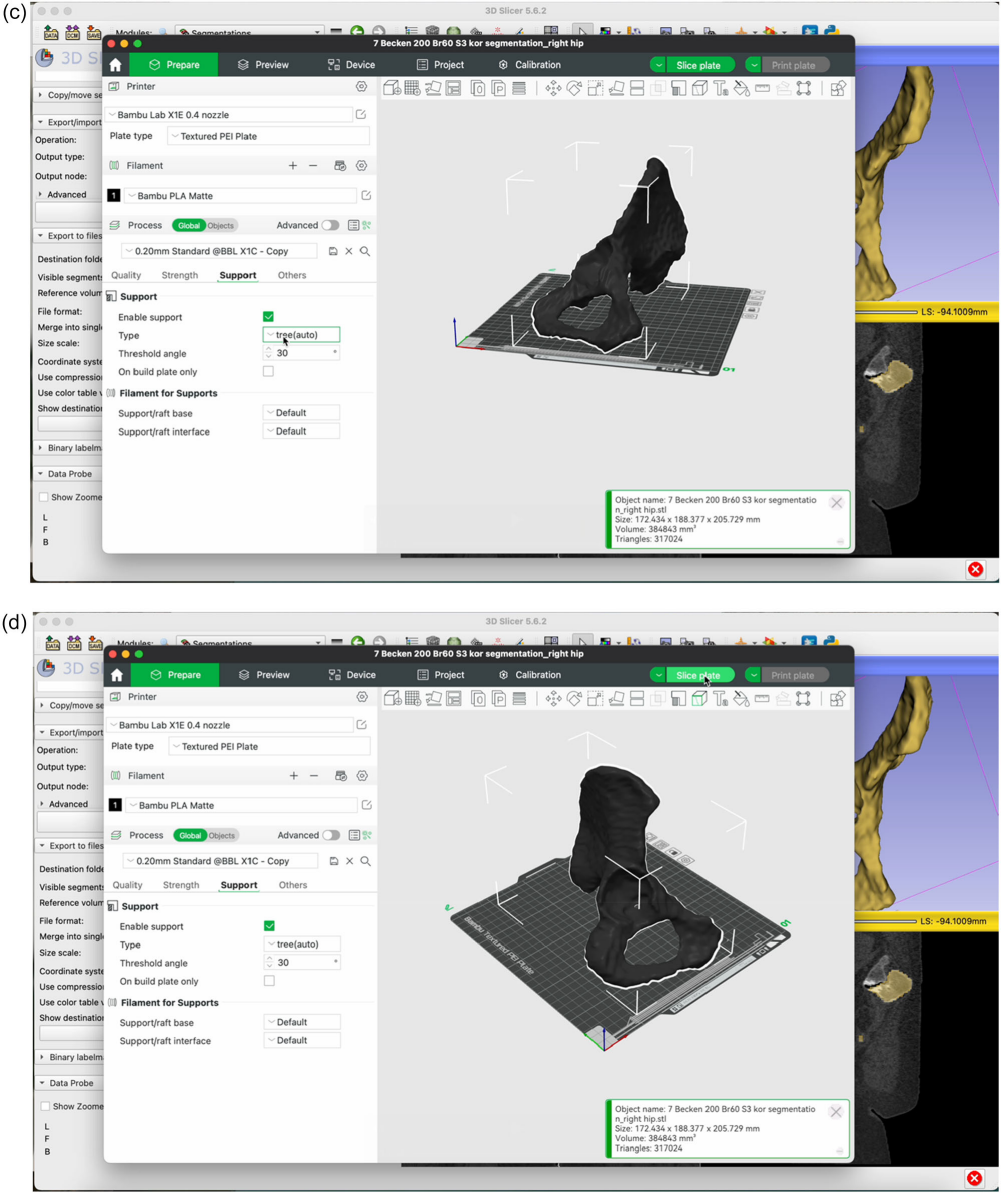

**Figure:**
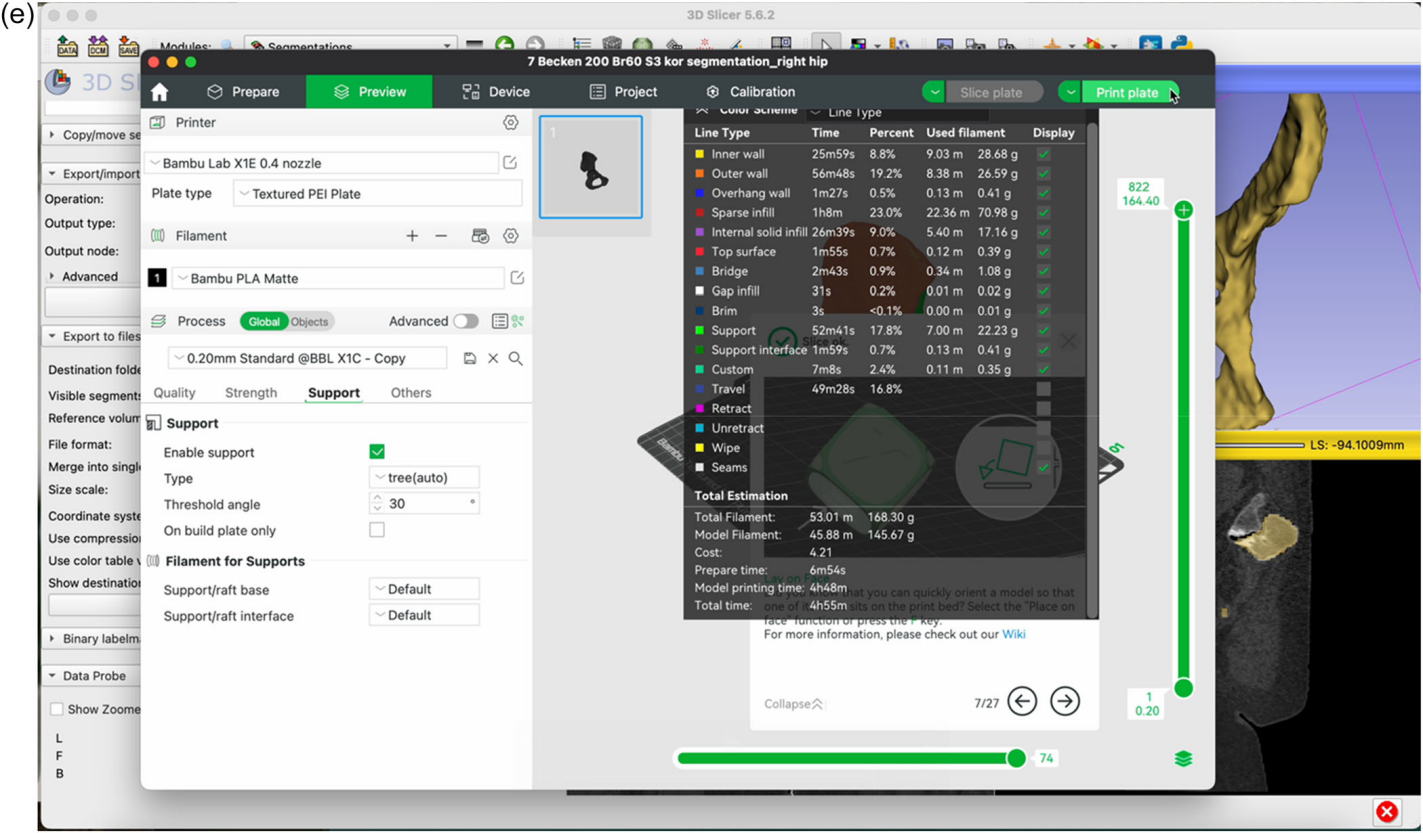

### AI use statement

A large language model (ChatGPT, OpenAI) was used to support language editing and text refinement during manuscript preparation. The authors reviewed and edited the content to ensure accuracy and accept full responsibility for the final manuscript. Generative AI did not contribute to data, results or analyses.

## RESULTS

**Figure 7:**
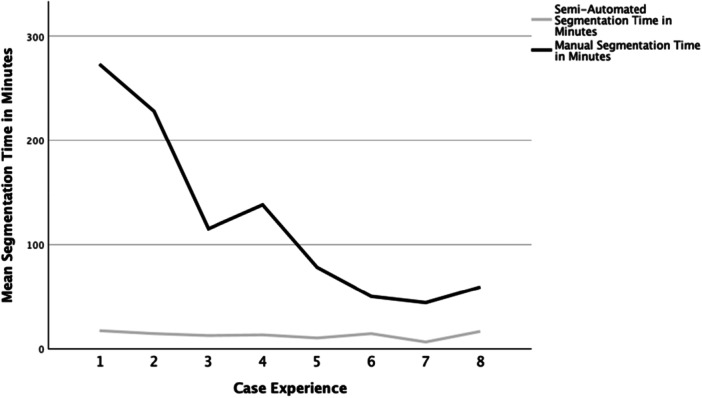
Comparison of the learning curve for the first eight models using either manual or semiautomated segmentation.

Manual segmentation time decreased from 273 min for the first case to a minimum of 44 min for the seventh case (mean: 123 min, standard deviation [SD] ± 86 min). The last three cases segmented manually were all performed in less than 1 h. For the eighth case, manual segmentation was performed in 59 min, and additionally, for the first time, our semiautomated workflow was applied. The resulting segmentation time decreased to 17 min. Mean segmentation time for the first eight cases using the semiautomated workflow was 13 min (SD ± 3 min), significantly shorter than manual segmentation (*p* = 0.003) (Figure 7).

**Figure 8:**
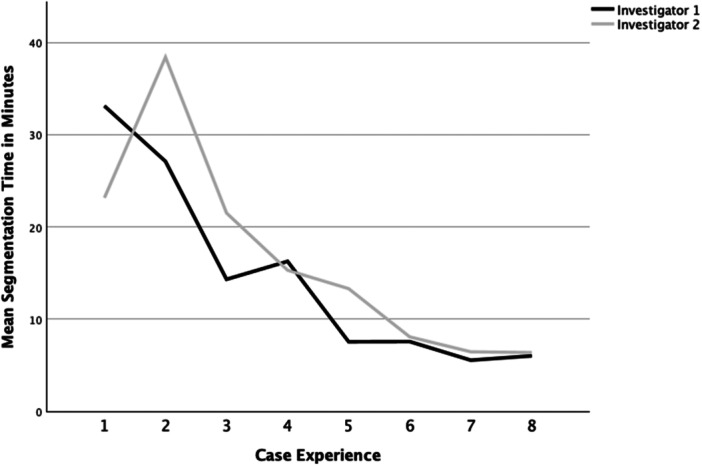
Mean segmentation times as performed by the two investigators for native hips.

Segmentation time for the native hips decreased from 33/23 min for the first case, to 6/6 min for the last case as performed by each investigator. The mean segmentation time was 15 min (SD ± 10 min), and there was no significant difference in mean segmentation time between the two investigators (*p* = 0.43). The last three segmentations were performed in less than 10 min each by both investigators (Figure 8). Application of a logarithmic regression model verified a strong learning effect in mean segmentation time for both investigators. 84.7% of the variance could be explained by the model (*R* ^2^ = 0.847, *p* = 0.001).

**Figure 9:**
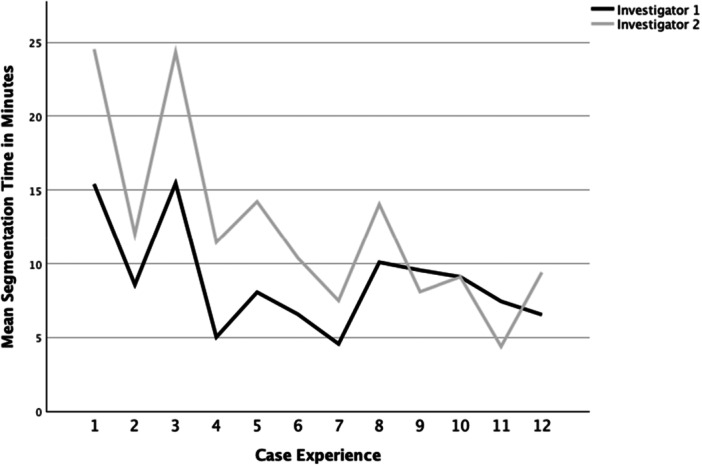
Mean segmentation times for pathologically altered hips.

Similarly, segmentation time for pathological cases decreased from 15/25 min for the first case, to 7/9 min for the last case performed by each investigator. The mean segmentation time for pathological cases was 11 min (SD ± 5 min). There was a significant difference between mean segmentation time for Investigator 1 (mean ± SD: 9 ± 4 min) and Investigator 2 (mean ± SD: 12 ± 6 min) with a *p* value of 0.007. The last three segmentations were performed in less than 10 min each by both investigators. Logarithmic regression analysis revealed a statistically significant (*p* = 0.007) model that explained 52.9% of the variance of the mean segmentation time (*R* ^2^ = 0.529). The learning curve plateaued after five cases for both native and pathological hips (Figure 9).

Mean filament weight used in the printing of the native hip models was 155.5 (SD ± 27.8) grams for primary cases, with no significant difference between the investigators (*p* = 0.063). For pathological hips, mean filament weight amounted to a mean of 155.5 (SD ± 27.0) grams with no significant differences between investigators (*p* = 0.802).

Mean printing time was 5 h (SD ± 1) for both native and for pathological hip models. Mean material cost per model was 3.88 euros (SD ± 0.70) for native hips and 3.90 euros (SD ± 0.65) for pathological models.

**Figure 10:**
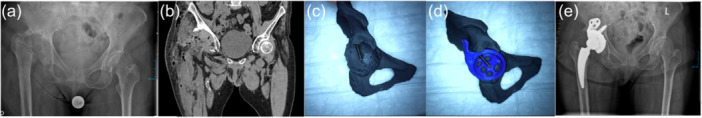
Plain pelvic radiograph (a), computed tomography (b), printed model and template trialling (c, d) after reaming including positioning of the iliac peg, radiograph after implantation (e) in a case featuring a Paprosky Type IIIa defect after periprosthetic infection and two‐stage revision.

According to the manufacturer, the Bamboo Lab H2D printer averages a power consumption of 0.197 kWh/h. With a printing time of 5 h, the overall power consumption amounts to 0.985 kWh. Assuming an electricity rate of 0.35 euros/kWh, the total energy costs for printing a pelvic model average around 0.34 euros. Prices for 3D printers have decreased over the last decade, currently ranging from several hundred to several thousand euros. A basic version of the printer used for the purpose of this study is commercially available for 1899 euros according to the manufacturer at the time of submission (August 2025). Figure 10 shows an exemplary patient case including preoperative radiographs, a model complete with trial implant and postoperative result.

**Figure 11:**
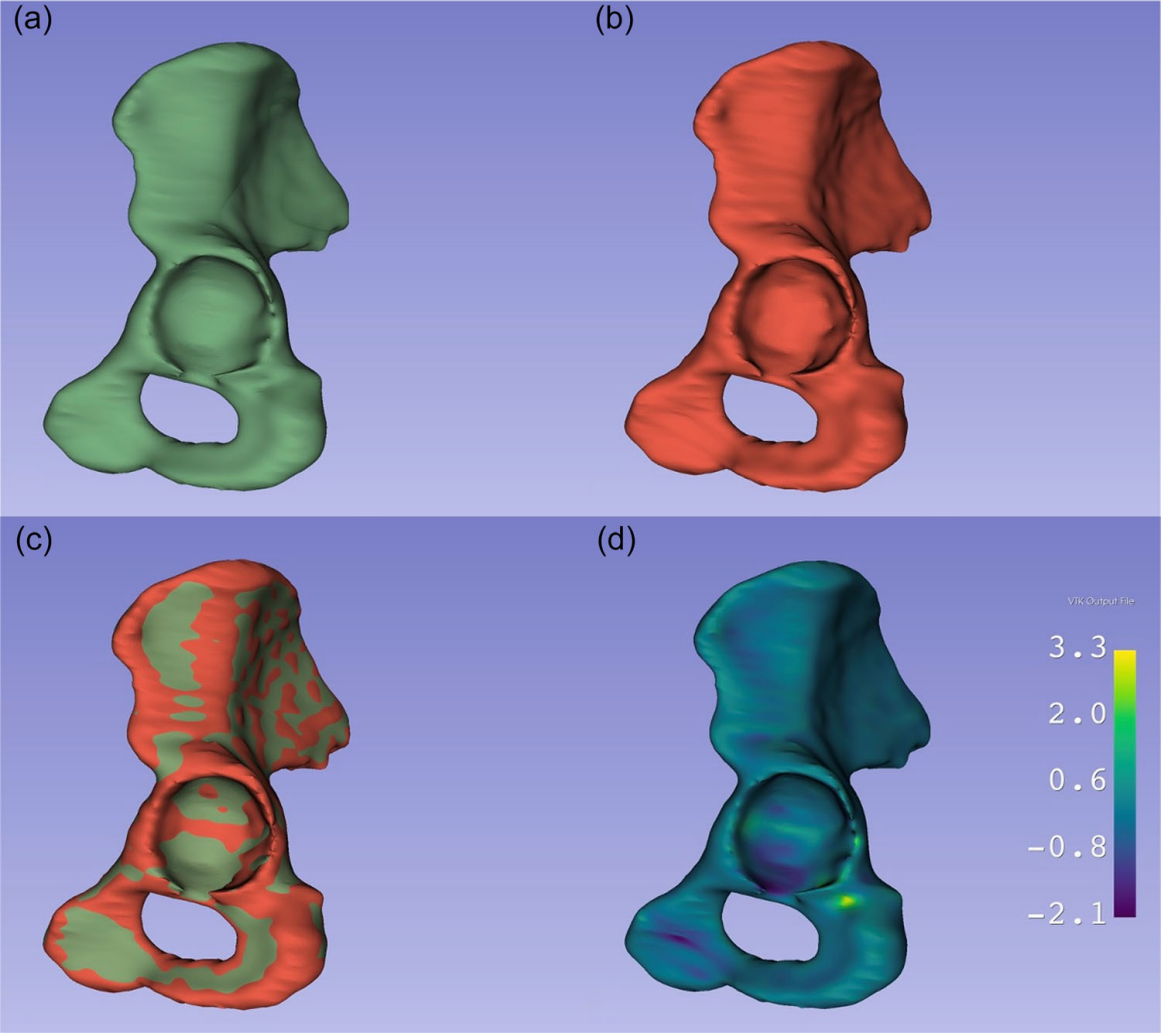
Exemplary comparison of segmentations by Investigator 1 (a), Investigator 2 (b), both segmentations merged into one (c) and a heatmap showing point‐to‐point surface distances in millimetres between both segmentations (d).

A virtual overlay of a model segmented by both investigators shows a near‐perfect match between both segmentations (Figure 11).

## DISCUSSION

In this study, we established a streamlined, semiautomated workflow for generating accurate, 3D printable models of the pelvis and acetabulum using only free and open‐source software. Our approach, centred around 3D Slicer and the TotalSegmentator module, demonstrated high usability and time efficiency, even for users with limited prior experience. The results support the feasibility of implementing this technique in routine orthopaedic practice—for both educational purposes and preoperative planning in primary and revision THA.

When comparing manual to semiautomated segmentation, the initial learning curve for manual segmentation is not representative, as the first steps using a new software are obviously the most time‐consuming. Nonetheless, the time for semiautomated segmentation in the eighth case was less than a third when directly compared to manual segmentation for the same model. This showcases that a semiautomated workflow is considerably less time‐consuming than traditional manual segmentation. Consecutively, the first ever case performed by both of our study participants took half the time of the last manually segmented case. Both study participants were able to master a steep learning curve, achieving segmentation times of under 10 min within the first 10 cases for both primary and pathological hips.

It is important to note that segmentation of pathologically altered hip models is considerably more complex than in native hip models. The main reason for this is that in any defect situation, excess bone has to be removed carefully from the model by manual refinement of the defect zone. This can be seen in Figure 9, where both investigators achieved similarly high or low segmentation times for the same respective cases, especially in cases one through eight, the graphs showing the segmentation time for each model are almost parallel.

Importantly, our learning curve analysis showed that two users with no prior experience regarding segmentation for 3D printing were able to independently complete the segmentation tasks with increasing efficiency, suggesting that the protocol is quickly learnable and robust across user backgrounds. These findings are in line with previous reports showing that simplified workflows reduce time burden without compromising anatomical accuracy [7]. An exemplary comparison of two models of the same hip as segmented by Investigators 1 and 2, respectively, as well as a merged segmentation and a heatmap showing point‐to‐point distances between the two segmentations showcases their quality (Figure 11).

Murphy and Wong published a step‐by‐step guide that allows the creation of 3D models in the context of femoral acetabular impingement and hip arthroscopy in 2021. It utilizes the freeware Horos (Nimble Co. LLC) and a thresholding technique that creates models based on Hounsfield units similar to our own workflow [9]. The described workflow is presumably comparable to our technique in terms of time and cost effectiveness. However, in the context of arthroplasty and especially revision arthroplasty, bone fragments and trabecular bone may be falsely labelled as part of the final model and need to be removed manually. Our technique allows a direct comparison of the segmentation with the CT scan and may be a more precise way of defining acetabular morphology compared to a thresholding technique with manual refinement of the final 3D model.

Prior studies have emphasized the value of 3D printing in improving implant positioning and preoperative accuracy in complex THA. Maryada et al. performed a preoperative surgical simulation using 3D models in 27 patients receiving either complex primary or revision THA and were able to predetermine the cup size used intraoperatively in 92.6% of cases [8]. Similarly, Zhang et al. were able to achieve a high agreement between planned and final cup size in 17 patients who underwent complex primary THA in the context of secondary arthritis due to developmental hip dysplasia and reported no complications at a mean follow‐up of 18 months. Furthermore, the authors were able to reliably restore the hip rotation centre [16]. Xu et al. compared two‐dimensional (2D) templating and 3D preoperative trialling in 14 cases where a THA was performed as a result of developmental dysplasia of the hip and were able to show a significantly higher correlation of planned and actual implant size with 3D planning. The final size was only determined in one case using 2D planning, while 3D planning correctly determined the final size in 10 cases [15]. Pflueger et al. were able to showcase the importance of accurate planning in the context of 3D‐printed patient‐specific instrumented total knee arthroplasty, reporting that the preoperative difference in 2D to 3D planning correlates with the difference between planned and achieved surgical correction [12]. While we did not analyse the level of anatomical detail of our models as a focus of this study, the fact that there was no significant difference in mean material consumption between the investigators leads us to the assumption that both investigators were able to create at least similarly accurate segmentations.

Jiang et al. showed that rapid in‐house printing of pelvic models usable for preoperative planning and trialling enables surgical intervention within 24 h of admittance to the hospital. The authors argue that in‐house printing of acetabular models poses a great advantage over outsourcing this to a third‐party in terms of time and cost effectiveness [6]. Our findings support this statement. With our workflow, same‐day surgery becomes viable even in complex cases.

While commercial, third‐party outsourcing solutions exist [14], they often rely on costly software, lengthy timelines, or steep learning curves, making them inaccessible for many institutions. In contrast, our workflow offers a cost‐effective and accessible alternative, which could lower the entry barrier for hospitals with limited resources or technical support. The costs for both native and pathologically altered models were well under 5 euros per model, even if the energy costs of printing the models are included. Of course, the overall cost of the respective models varies depending on the printer and the material used. Murphy and Wong report printing costs of about 5–7 US dollars per print using PLA filament [9]. Xu et al. postulated costs of about 400 US dollars per print [15], while Jiang et al. directly compared costs of three different kinds of material. A model consisting of nylon cost 100 dollars, plaster 250 dollars and a resin model amounted to 1200 US dollars [6]. Our results are highly economical, the costs of the printer as well as the material costs may well be considered negligible when compared to overall perioperative costs regarding surgical staff, logistics and implants.

While the primary focus of this study was on developing and validating the technical and financial feasibility of the workflow, the models produced could be leveraged in various clinical contexts. For instance, patient‐specific anatomical templates can support decisions about whether standard, off‐the‐shelf implants are sufficient, or whether a case might benefit from a custom 3D‐printed component. This is particularly relevant in revision settings, where acetabular bone loss is common and the availability of suitable implants can be unpredictable. As such, this workflow could aid surgeons and institutions in anticipating implant requirements and potentially streamline logistics by reducing the need for intraoperative decision‐making or urgent implant sourcing.

Moreover, the ability to produce printable models on‐site may enable same‐day planning or simulation, providing a faster turnaround than most third‐party services. This could be particularly valuable for hospitals without access to specialized implant libraries or for cases requiring rapid planning decisions.

## LIMITATIONS

This study does not include clinical outcome data and, therefore, does not assess whether the use of these models improves surgical accuracy or patient outcomes. However, our intent was to validate the technical and financial feasibility, accessibility, and efficiency of the workflow itself. Further studies are needed to assess its clinical utility, including comparisons of surgical time, implant accuracy, complication rates, and patient‐reported outcomes in prospective cohorts. The surgeons who used the models at our institution suggested that the haptic feedback achieved by handling the 3D models enhances surgical planning and that the opportunity to simulate the surgery in a hands‐on setting poses additional value even when digital 3D planning software is available. This is, of course, speculative and further studies are needed to verify this assessment.

## CONCLUSIONS

We presented a time and cost‐effective workflow for the manufacturing of pelvic models that is easily adaptable and may potentially help enhance surgical outcomes in primary and revision hip arthroplasty. Future research should also explore how this workflow could be scaled across institutions and adapted to other anatomical regions. Ultimately, the use of semiautomated 3D printing workflows may contribute to more personalized, efficient and resource‐conscious orthopaedic care.

## AUTHOR CONTRIBUTIONS

**Hendrik Pott, Stephan Brand and Max Ettinger:** Conceptualization. **Hendrik Pott, Peter Savov and Ricarda Stauss:** Methodology. **Hendrik Pott, Julian‐Arman Beheshty and Felix Thormann:** Investigation. **Hendrik Pott and Ricarda Stauss:** Data curation. **Hendrik Pott:** Writing—original draft. **All authors:** Writing—review and editing. All authors have read and revised the manuscript.

## CONFLICTS OF INTEREST STATEMENT

Peter Savov is an instructor for Smith and Nephew. Max Ettinger is an instructor and consultant for Smith and Nephew and Microport. The remaining authors declare no conflict of interest.

## ETHICS STATEMENT

All procedures performed were in accordance with the ethical standards of the responsible committee on human experimentation (institutional and national) and with the Helsinki Declaration of 1975 (in its most recently amended version). Informed consent was obtained from all patients for whom identifying information is included in this article.

## Data Availability

The authors have nothing to report.
